# Differential Factors Associated With the Presence of Persistent Symptoms in Individuals Diagnosed With Long COVID: Protocol for a Longitudinal Matched Case-Control Study

**DOI:** 10.2196/67133

**Published:** 2026-03-24

**Authors:** David Lerma-Irureta, Fátima Méndez-López, Elena Navarro-Matías, Diego Lerma-Puertas, Rosa Magallón-Botaya

**Affiliations:** 1 Department of Medicine, Psychiatry and Dermatology University of Zaragoza Zaragoza, Spain Zaragoza Spain; 2 Instituto de Investigación Sanitaria Aragón Zaragoza Spain; 3 Universidad de Zaragoza Faculty of Health Sciences Department of Physiatry and Nursery Zaragoza Spain; 4 Instituto de Investigación Biomédica de Salamanca Salamanca Spain; 5 Obstetrics Department Hospital Clínico Universitario Lozano Blesa Zaragoza Spain; 6 Department of Surgery, Gynecology, and Obstetrics Universidad de Zaragoza Zaragoza Spain

**Keywords:** biomarkers, cohort study, lifestyle factors, Long COVID, machine learning, persistent symptoms, postacute sequelae of SARS-CoV-2 infection, quality of life

## Abstract

**Background:**

Long COVID (postacute sequelae of SARS-CoV-2 infection) is a heterogeneous condition with persistent multisystem symptoms and substantial functional burden. Integrative longitudinal studies combining clinical phenotyping, lifestyle factors, and immunobiological markers are needed to clarify determinants of symptom persistence and inform risk stratification and targeted interventions.

**Objective:**

This study aims to identify clinical, biological/immunological, and sociodemographic factors associated with Long COVID status by comparing individuals with persistent symptoms to matched recovered controls, and to evaluate longitudinal changes in symptoms and secondary outcomes over follow-up.

**Methods:**

ARALongCOV is a longitudinal matched case-control observational study conducted in Aragón, Spain, including adults (≥18 years of age) with confirmed SARS-CoV-2 infection. Participants are recruited through 3 sources: the Long COVID Aragón Patient Association, the Aragón Health Service database, and primary care consultations. Long COVID and recovered participants are individually matched 1:1 (without replacement) on sex/gender (exact), age (±3 years), and date of acute COVID-19 diagnosis (±30 days). Outcomes include persistent symptoms and functioning and patient-reported outcomes (quality of life, physical activity, diet, sleep, mental health, functional status, cognitive performance, pain catastrophizing, and fatigue), alongside clinical variables and biochemical/immunological markers (including inflammatory and cytokine profiles, SARS-CoV-2 antispike immunoglobulin G serology, and viral reactivation serologies). Measurements are obtained at baseline (T0) and repeated at follow-up (T1) using standardized procedures.

**Results:**

The study received ethics approval from the Clinical Research Ethics Committee of Aragón (PI21/278). Funding was provided by Instituto de Salud Carlos III through project PI22/01070 (cofunded by the European Union) for the period 2023-2027. Baseline assessments (T0) were initiated in late 2022/early 2023. As of February 2026, a total of 200 participants have been enrolled (n=100 Long COVID; n=100 recovered controls) and have completed T0; T1 assessments are scheduled for late 2025/early 2026 (~3-year follow-up for the earliest enrolled participants). Primary analyses will be conducted after completion of T1 assessments, with dissemination planned from the second half of 2026 and continuing through 2027.

**Conclusions:**

This protocol describes a comprehensive, multidimensional longitudinal study designed to clarify determinants of Long COVID by integrating clinical, functional, lifestyle, and immunobiological data in matched cohorts. Findings are expected to support risk stratification, phenotype discovery, and identification of prognostic markers to inform preventive, diagnostic, and rehabilitative strategies.

**Trial Registration:**

ISRCTN Registry ISRCTN27312680; https://tinyurl.com/33cbysrk

**International Registered Report Identifier (IRRID):**

DERR1-10.2196/67133

## Introduction

### Overview

The COVID-19 pandemic has substantially reshaped the global health burden by producing not only acute morbidity and mortality but also a spectrum of persistent postinfectious sequelae that continue to affect large numbers of survivors [1-3]. One of the most clinically and socioeconomically consequential of these sequelae is Long COVID, frequently termed postacute sequelae of SARS-CoV-2 infection (PASC), a heterogeneous syndrome comprising persistent, relapsing, or newly emergent symptoms after the acute phase of infection [4-6]. Population and cohort analyses show appreciable heterogeneity in estimates of prevalence across definitions, follow-up intervals, and dominant variants, with many studies reporting persistent symptoms in the approximate 10%-30% range among infected persons depending on case definition and follow-up timing [7-9]. Typical symptom clusters include debilitating fatigue, dyspnea/exertional intolerance, and cognitive dysfunction (often described as “brain fog”), and these complaints occur in individuals across the spectrum of acute disease severity, including those who had mild acute illness [2,8,9].

Mechanistic concepts for PASC are multifactorial and emphasize an interplay of viral, immune, and host-environment factors, including potential viral persistence or residual antigen, dysregulated or protracted immune activation, chronic systemic and tissue-specific inflammation, perturbations of metabolic pathways, and, in some patients, autoimmune sequelae [5,10,11]. Proteomic and metabolomic studies and longitudinal immune profiling have reported persistent alterations in circulating proteins and immune mediators—including reported associations with proinflammatory cytokines such as interleukin-6 (IL-6) and tumor necrosis factor-α—although results vary across cohorts and time points [10,12,13]. At the same time, psychosocial determinants (eg, preexisting psychiatric morbidity, chronic stress, and social isolation) and baseline comorbidity burden (eg, obesity, cardiopulmonary disease, and chronic kidney disease) have been associated with greater acute severity and may influence recovery trajectories, symptom perception, and care seeking after SARS-CoV-2 infection [14-17].

Despite rapid expansion of descriptive and mechanistic work, the extant literature remains fragmented: many investigations target single domains (immunologic, imaging, symptom inventories, or psychosocial measures) or selected subpopulations (hospitalized cases, clinic-referred patients, or pediatric cohorts), limiting integrative inference and generalizability [5,18]. Large electronic health record–driven and population-based studies have mapped cooccurrence patterns and identified candidate risk factors, but frequently lack prospectively collected multimodal biomarker and psychosocial data required to link biological signals to clinically meaningful longitudinal trajectories [6,19]. Neurocognitive sequelae exemplify this gap: observational and translational work implicates persistent neuroinflammation and systemic immune perturbations as plausible contributors to post–COVID-19 cognitive complaints and to accelerated decline among susceptible individuals, but definitive, integrative longitudinal evidence is limited [20-22]. Taken together, these observations emphasize the need for prospective, multimodal cohorts that combine standardized clinical phenotyping, serial biosampling, and structured psychosocial measurement to permit hypothesis-driven biomarker discovery and etiologic modeling of PASC [3,23].

Demographic factors and baseline comorbidities modulate both acute disease course and postacute outcomes: older age and multimorbidity are consistently associated with a higher risk of persistent symptoms and adverse long-term outcomes in many cohort studies, and prior cognitive impairment has been identified as a vulnerability factor for prolonged neuropsychiatric sequelae [9,24-26]. Obesity and chronic respiratory disease have been linked to greater acute severity and higher likelihood of prolonged symptoms, and these associations have implications for targeted surveillance and rehabilitation strategies [17,27,28]. Collectively, these epidemiologic and mechanistic observations underscore the need for health systems to develop evidence-based approaches to identify at-risk populations early and to tailor prevention, monitoring, and rehabilitation resources accordingly [3,23,29].

In summary, although the body of research on Long COVID has expanded quickly, there remains an urgent requirement for coordinated, integrative studies that synthesize clinical phenotyping, longitudinal immunology, objective physiologic assessment, and psychosocial characterization, with the ultimate goal of deriving reproducible biomarkers, mechanism-informed subtypes, and actionable interventions for PASC [3,5,6,12,23].

### Hypothesis and Rationale

We hypothesize that antecedent and periacute factors (eg, sociodemographic, preinfection comorbidities, and acute COVID-19 severity indicators) are associated with Long COVID status, and that baseline postacute measures collected at T0 (eg, patient-reported outcome measures and biochemical/immunological markers) primarily differentiate Long COVID from recovered controls as concurrent phenotypic correlates rather than implying causality. By comparing individuals with Long COVID to carefully matched recovered controls and repeating standardized assessments longitudinally, we aim to identify differential factors across clinical, immunological, and lifestyle domains. These findings are expected to support biomarker discovery, mechanism-informed subtyping, and clinically actionable risk stratification in future studies.

### Study Objectives

#### Primary Objective

The primary objective is to estimate associations between antecedent/periacute clinical and sociodemographic factors and Long COVID status, and to characterize postacute T0 clinical, biomarker, and psychosocial profiles as phenotypic differentiators between cases and matched recovered controls.

#### Secondary Objectives

The secondary objectives are as follows:

1. To characterize the Long COVID cohort with respect to demographics, comorbidity burden, and lifestyle habits.
2. To evaluate associations between lifestyle factors and preexisting conditions and the presence and severity of persistent symptoms and functional impairment.
3. To examine longitudinal change in symptoms and patient-reported outcomes between T0 and T1 within each group and compare trajectories between groups.

This protocol therefore describes a longitudinal matched case-control study designed to integrate clinical, functional, lifestyle, and immunobiological data to better understand determinants of persistent post–COVID-19 symptoms.

## Methods

### Study Design

This study is a longitudinal matched case-control study design combining a retrospective cohort with a prospective cohort. The retrospective cohort comprises individuals diagnosed during the early stages of the pandemic and provides long-term follow-up. In parallel, the prospective cohort will enroll newly identified COVID-19 cases, with follow-up assessments conducted after the baseline evaluation. This combined approach allows examination of symptom persistence for up to 3 years in the retrospective cohort while also enabling real-time tracking of changes over time in the prospectively enrolled participants. The study is registered in the International Standard Randomized Controlled Trial Number (ISRCTN) Registry (ISRCTN27312680), and reporting will adhere to the STROBE (Strengthening the Reporting of Observational Studies in Epidemiology) guidelines.

### Study Setting and Population

The study will be conducted in Aragón, Spain, leveraging the region’s established primary care network and health care infrastructure. Eligible participants will be adults (≥18 years of age) with a confirmed diagnosis of COVID-19. Participants will be identified through three complementary sources: (1) the Long COVID Aragón Patient Association (patients already registered with the association), (2) the Aragón Health Service database (individuals identified from electronic health records), and (3) primary care consultations (direct referrals from general practitioners and other health care professionals).

The Long COVID Aragón Patient Association is a regional, patient-led organization based in Aragón, Spain, that provides peer support, advocacy, and information for individuals experiencing persistent symptoms following SARS-CoV-2 infection. The association was established in 2020 in response to the emergence of postacute sequelae of COVID-19 and maintains a secure registry of members who voluntarily enroll and provide contact information and basic clinical descriptors (eg, date of acute infection, persistence and type of symptoms, and care pathways). Members who indicate willingness to be contacted for research constitute an important recruitment frame for this study, enabling identification of community-dwelling individuals with persistent symptoms who may not be fully captured through health care databases alone. For eligible individuals recruited through the association, study participation will proceed via the same standardized eligibility assessment and consent procedures used for all recruitment sources, and (where applicable and permitted) self-reported information will be verified or complemented using clinical records.

### Eligibility Criteria

Participants will be included if they have confirmed SARS-CoV-2 infection by polymerase chain reaction, antigen testing, or serology, and are aged ≥18 years. For the Long COVID group, eligibility will require persistence of symptoms for ≥12 weeks after infection onset, consistent with the World Health Organization (WHO) definition. For the control group, complete recovery will be defined as the absence of persistent or new symptoms after COVID-19. Individuals will be excluded if they refuse to participate, report preexisting symptoms similar to Long COVID prior to SARS-CoV-2 infection, or present severe psychiatric disorders or cognitive impairment that preclude informed participation.

### Sample Size Calculation

Because the primary aim of this study is hypothesis-generating—that is, to identify differential factors associated with Long COVID status rather than to test a single prespecified end point—sample size was chosen to provide adequate power to detect moderate associations between candidate predictors and case status within a matched case-control framework. Power was evaluated using the standard normal approximation for comparing 2 independent proportions (Fleiss et al [30]), with a 2-sided significance level of α=.05 and 90% power. Under a conservative scenario in which a candidate factor has an expected prevalence of 20% among controls, we considered an association of odds ratio 2.3 to be clinically meaningful; this corresponds to an expected prevalence of approximately 36.5% among cases. Under these assumptions, the required analyzable sample size is approximately 154 participants per group, rounded to 155 per group. To accommodate incomplete matching, potential attrition between T0 and T1, and planned multivariable and subgroup analyses, the overall recruitment target will be increased to approximately 400 participants [30].

### Matching Strategy

Recovered controls will be individually matched 1:1 to Long COVID cases without replacement to improve baseline comparability. Matching will require exact matching on sex/gender, with flexibility of ±3 years for age and ±30 days for date of acute COVID-19 diagnosis. When multiple eligible controls are available for a given case, the selected control will be the one minimizing the absolute age difference; if ties remain, the absolute diagnosis date difference will be minimized, with random selection used only if ties persist. Each matched set will be assigned a unique pair identifier (pair_id) and retained in the analytical dataset; participants who cannot be matched within the prespecified calipers will be retained in the study database and considered in prespecified sensitivity analyses.

### Data Collection and Management

After written informed consent is obtained, each participant will be assigned a unique internal study code to ensure confidentiality. The code key linking personal identifiers to the study code will be accessible only to the principal investigator and will be stored separately from study data, in accordance with the General Data Protection Regulation (GDPR) and applicable Spanish data protection legislation. All study information will be recorded and managed using secure electronic data capture procedures; self-administered questionnaires will be completed via REDCap (Research Electronic Data Capture; Vanderbilt University) software [31]. Study datasets will be deidentified/anonymized for analysis and will be stored securely, with participants linked only through the assigned study code.

### Appointments and Procedures

**Figure 1:**
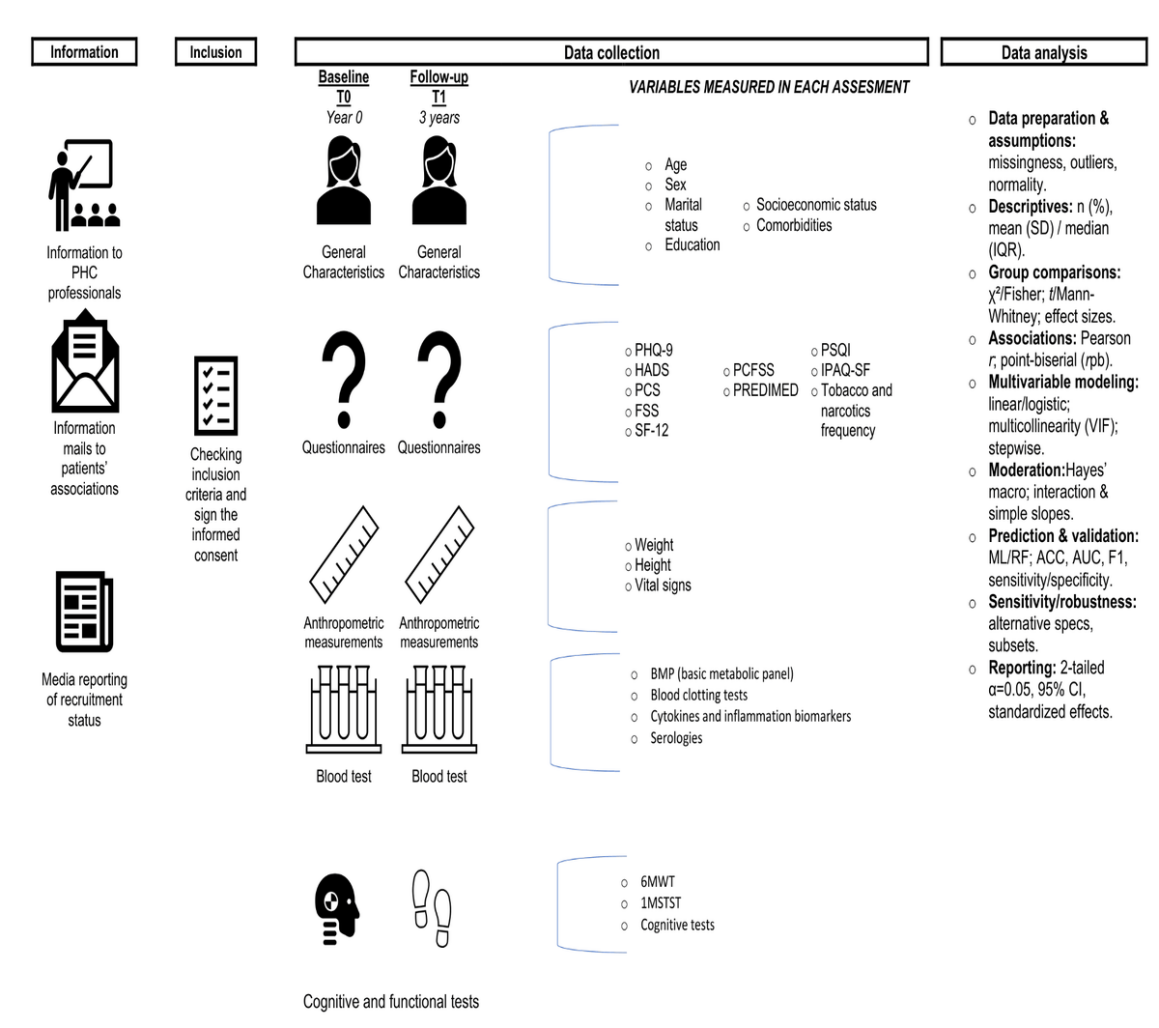
Participant flow diagram. 1MSTST: 1-minute sit-to-stand test; 6MWT: 6-minute walk test; ACC: accuracy; AUC: area under the receiver operating characteristic curve; FSS: Fatigue Severity Scale; HADS: Hospital Anxiety and Depression Scale; IPAQ-SF: International Physical Activity Questionnaire-Short Form; ML: machine learning; PCFSS: Post–COVID-19 Functional Status Scale; PCS: Pain Catastrophizing Scale; PHC: primary health care; PHQ-9: Patient Health Questionnaire-9; PREDIMED: Prevención con DIeta Mediterránea; PSQI: Pittsburgh Sleep Quality Index; RF: random forest; SF-12: 12-Item Short Form Health Survey.

Data collection will be organized into 2 study appointments. During the first appointment, fasting blood samples will be collected for routine laboratory analyses, and participants will sign informed consent covering both study participation and biobank sample storage. Participants will also receive standardized instructions for completing the self-administered questionnaires online through REDCap [31]. The second appointment will include completion of exploratory tests and the collection of any additional clinical or sociodemographic data not captured at the first visit. Standardized procedures will be used at each visit to minimize measurement variability and ensure comparability across participants and across time points. The participant flow and assessment schedule are summarized in Figure 1.

### Variables and Measurements

#### Overview

Study variables will include outcomes related to persistent symptoms and functioning, additional patient-reported outcomes, cognitive performance measures, and laboratory biomarkers, alongside core sociodemographic and clinical covariates. Measurements will be obtained at T0 (baseline) and repeated at T1 (follow-up) using the same instruments and procedures to support longitudinal comparisons. Textbox 1 summarizes the study variables collected for sociodemographic, clinical, lifestyle, psychological/functional, and biological domains.

**Study variables.**

**Sociodemographic**

- Age, sex/gender, place of residence, occupation, education level, and ethnicity

**Clinical (acute COVID-19 episode)**

- Date of symptom onset, symptom profile, hospitalization, intensive care unit admission, and diagnostic method (polymerase chain reaction, antigen, and serology)

**Comorbidities**

- Cardiovascular disease, diabetes mellitus, chronic respiratory disease, and other chronic conditions

**Lifestyle**

- Physical activity level (International Physical Activity Questionnaire), dietary habits (Prevención con Dieta Mediterránea), sleep quality (Pittsburgh Sleep Quality Index), smoking status, and alcohol consumption

**Psychological and functional**

- Quality of life (12-item Short Form Health Survey), depression (Patient Health Questionnaire-9), anxiety (Generalized Anxiety Disorder-7), functional status (Post–COVID-19 Functional Status Scale), cognitive flexibility and attention (Stroop Test), processing speed (Symbol Digit Modalities Test), visuospatial memory (Rey-Osterrieth), pain catastrophizing (Pain Catastrophizing Scale), and fatigue severity (Fatigue Severity Scale)

**Biological**

- Complete blood count, basic biochemistry, coagulation profile, inflammatory markers (C-reactive protein and interleukin-6), cytokine profile, and SARS-CoV-2 serology

#### Primary Outcome

The primary outcome will be Long COVID status (case vs recovered control). Cases will be defined as individuals with confirmed SARS-CoV-2 infection who report persistent symptoms for ≥12 weeks after infection onset, consistent with the WHO definition. Controls will be defined as individuals with a confirmed SARS-CoV-2 infection who report complete recovery, operationalized as the absence of persistent or new symptoms after COVID-19.

#### Secondary Outcomes

Secondary outcomes will encompass patient-reported and performance-based domains including health-related quality of life, physical activity, dietary pattern/adherence, sleep quality, depressive and anxiety symptoms, post–COVID-19 functional status, cognitive performance, as well as pain catastrophizing, fatigue severity, and laboratory biomarkers (biochemistry/hematology/coagulation, inflammatory markers including C-reactive protein [CRP] and IL-6, cytokine profiling, SARS-CoV-2 serology, and viral reactivation serologies such as Epstein-Barr virus [EBV], cytomegalovirus, and herpes simplex virus type 1 [HSV-1]). The presence and severity of persistent symptoms consistent with Long COVID (eg, fatigue, dyspnea, and cognitive complaints), will be operationalized using standardized symptom assessment procedures within the Long COVID group and contrasted with the absence of persistent symptoms in the control group. Symptom severity will be quantified using the prespecified instruments and scales described in the “Sociodemographic and Clinical Variables” section, enabling both between-group comparisons at each time point and within-participant change from T0 to T1.

These outcomes will be analyzed as complementary indicators of disease burden and functioning and as potential correlates of persistent symptom status and its evolution over time.

#### Sociodemographic and Clinical Variables

Key sociodemographic variables will include age, sex/gender, place of residence, occupation, education, and ethnicity. Clinical history related to the acute SARS-CoV-2 infection will include onset date, acute symptoms, hospitalization, and intensive care unit (ICU) admission (if applicable), and the method used for diagnostic confirmation. Preexisting and incident comorbidities will be recorded, including (but not limited to) cardiovascular disease, diabetes, chronic respiratory disorders, and other relevant conditions, to support confounding control and stratified analyses.

#### Lifestyle and Psychosocial Variables

##### Overview

All participant-reported outcomes will be collected at the baseline visit (T0) and repeated at the follow-up visit (T1; ~3 years after T0) using the same administration procedures. Self-administered questionnaires were completed in a standardized order, with staff available to resolve comprehension issues without coaching responses. Neurocognitive tests were administered by trained personnel following published manuals and standardized instructions. A detailed summary of instruments, scoring, recall periods, and key measurement properties is provided in Multimedia Appendix 1 [32-47].

##### Health-Related Quality of Life

Health-related quality of life was assessed using the Spanish 12-item Short Form Health Survey (SF-12; a 12-item short-form measure derived from the 36-item Short Form Health Survey [SF-36]) that captures 8 health domains and yields 2 standardized summary scores: the physical component summary and mental component summary. Scores are computed using Spanish population weights and interpreted against population norms, with higher scores indicating better health status. In the Spanish validation work, the SF-12 showed acceptable metric performance and construct validity; notably, SF-12 items explained approximately 91% of the variance of SF-36 summary components, supporting the adequacy of the short form for estimating physical component summary and mental component summary in Spanish samples. Internal consistency for SF-12 summary measures has been reported in an acceptable-to-good range in Spain (with component reliability typically around α≈.78-.85) [32].

##### Physical Activity

Physical activity over the preceding week was measured using the International Physical Activity Questionnaire (IPAQ). The IPAQ quantifies time spent in activities of different intensities during the last 7 days, allowing the calculation of energy expenditure (eg, metabolic equivalent of task minutes per week) and categorical classifications of activity level. In the Spanish population, the IPAQ demonstrated good reproducibility and expected validity patterns: test-retest reliability was strong (Spearman ρ≈0.81; intraclass correlation coefficient [ICC]≈0.74), while criterion validity versus objective accelerometry was modest (approximately ρ≈0.27) and improved when compared with activity diaries (approximately ρ≈0.38), consistent with typical performance of self-reported physical activity measures [33].

##### Adherence to the Mediterranean Diet

Mediterranean diet adherence was assessed with the 14-item Mediterranean Diet Adherence Screener (MEDAS) developed within the Prevención con Dieta Mediterránea (PREDIMED) framework. The MEDAS is a brief checklist covering key Mediterranean diet targets; responses are summed to a total score from 0 to 14, with higher scores indicating greater adherence. As a dietary pattern screener (rather than a unidimensional psychometric scale), “validity” is primarily supported through its relationship with external criteria: in PREDIMED, the 14-item tool was used alongside a full-length food frequency questionnaire and showed a strong, graded association with obesity-related indices, supporting its capacity to capture meaningful differences in diet quality in high-risk adults [34].

##### Sleep Quality

Sleep quality was measured using the Pittsburgh Sleep Quality Index (PSQI), a 19-item questionnaire assessing sleep during the previous month and yielding 7 component scores (subjective sleep quality, latency, duration, efficiency, disturbances, sleep medication use, and daytime dysfunction) and a global score ranging from 0 to 21; higher scores indicate worse sleep quality. The original PSQI demonstrated strong clinical use and has been widely used in research settings [35]. In the Spanish version, clinometric evaluation supports its use, with internal consistency around α≈0.81 and evidence of diagnostic performance using standard thresholds (with sensitivity in the high 80% range and specificity around the mid-70% range, depending on the criterion) [36].

##### Depressive Symptoms

Depressive symptom severity was assessed with the Patient Health Questionnaire-9 (PHQ-9), a 9-item self-report measure aligned with the *DSM* (*Diagnostic and Statistical Manual of Mental Disorders*) symptom criteria and anchored to the preceding 2 weeks. Each item is scored 0 (“not at all”) to 3 (“nearly every day”), producing a total score from 0 to 27, where higher scores indicate more severe depressive symptoms. In Spanish primary care validation, the PHQ-9 showed strong internal consistency (reported as McDonald ω≈0.89) and good screening performance for major depressive disorder, with high sensitivity and moderate specificity depending on the selected cut-point and reference standard [37].

##### Anxiety Symptoms

Anxiety symptom severity was measured with the Generalized Anxiety Disorder-7, a 7-item self-report scale also anchored to the preceding 2 weeks, using the same 0-3 response options and yielding a total score from 0 to 21; higher scores indicate more severe anxiety. The Spanish adaptation demonstrated excellent internal consistency (α≈0.94) and strong short-term stability (test-retest ICC≈0.84), alongside evidence of concurrent validity through strong correlations with established anxiety and disability measures [38].

##### Post–COVID-19 Functional Status Scale

Functional limitations were assessed using the Post–COVID-19 Functional Status Scale (PCFS), administered as a self-completed web-based questionnaire (REDCap). The PCFS is an ordinal measure grading limitations from 0 (no functional limitations) to 4 (severe functional limitations), with higher grades indicating greater restriction in usual activities and lifestyle. Because PCFS is an ordinal functional status grade rather than a multi-item psychometric scale, internal consistency is not applicable; instead, measurement quality is supported by agreement and construct validity [39]. The Spanish web-based PCFS has demonstrated substantial test-retest reliability (κ=0.63) and high agreement between web- and paper-based formats (88% agreement), alongside strong construct validity through expected correlations with health status and activity limitation measures (eg, EQ-5D-5L questionnaire and Global Activity Limitation Indicator) [40].

##### Cognitive Performance

Cognitive domains relevant to attention/executive control, processing speed, and visuoconstructive memory were assessed with standardized neuropsychological tests. Selective attention and inhibitory control were assessed using the Stroop Color and Word Test, generating condition scores and an interference index reflecting executive control under competing stimulus demands [41]. Processing speed and attention were assessed with the Symbol Digit Modalities Test, which measures rapid visual scanning, sustained attention, and speeded substitution under timed conditions [42]. Visuoconstruction and visual memory were evaluated using the Rey-Osterrieth Complex Figure, including copy accuracy and recall performance using standardized scoring procedures [43]. These instruments have extensive normative use in clinical and experimental contexts, and standardized administration enhances reliability across assessment waves.

##### Pain Catastrophizing

Maladaptive pain-related cognition was measured with the Pain Catastrophizing Scale (PCS), a 13-item measure covering rumination, magnification, and helplessness. Items are typically rated on a 0-4 scale and summed to a total score from 0 to 52, where higher scores reflect greater catastrophizing. The original PCS demonstrated robust psychometric development and validation [44], and the Spanish version has shown excellent internal consistency (α≈.94) and strong test-retest reliability (ICC≈0.88), supporting its use in Spanish-speaking clinical populations [45].

##### Fatigue Severity

Fatigue was assessed using the Fatigue Severity Scale (FSS), a 9-item questionnaire capturing the impact and severity of fatigue on daily functioning. Responses are typically scored from 1 to 7 and summarized as a mean score (commonly interpreted with higher values indicating greater fatigue severity) [46]. In post–COVID-19 recovery clinic populations, the FSS showed excellent internal consistency (α≈0.96) and expected construct validity through correlations with quality of life and depressive symptoms, supporting its use for quantifying clinically relevant fatigue in this context [47].

#### Biochemical and Immunological Markers

Fasting venous blood samples will be collected at baseline (T0) and follow-up (T1) to characterize participants’ biochemical, hematological, coagulation, inflammatory, and immunological profiles. All measurements will be performed in the routine hospital clinical laboratory according to standard operating procedures. Standard panels will include basic biochemistry, a complete blood count, and a coagulation profile. Systemic inflammation will be assessed using circulating inflammatory markers, including CRP and IL-6, and broader immune signatures will be characterized through cytokine profiling using a predefined panel. Humoral response to SARS-CoV-2 will be assessed using quantitative antispike immunoglobulin G serology, recorded as a continuous measure. Potential viral reactivation will be explored by measuring serological markers for latent herpesviruses, including EBV, cytomegalovirus, and HSV-1. Biomarker variables will be analyzed as candidate correlates of persistent symptoms and as longitudinal measures to evaluate change between T0 and T1.

### Statistical Analysis

#### Overview

All analyses will be performed using IBM SPSS Statistics (version 27) and R software (R Foundation for Statistical Computing). Statistical inference will emphasize effect sizes with 95% CIs and clinically interpretable magnitudes; 2-sided tests will be used. Given the observational design, analyses will be interpreted as associations rather than causal effects. For interpretability, candidate “differential factors” will be grouped by temporality: (1) antecedent factors (eg, sociodemographic and preinfection comorbidities), (2) periacute factors (eg, markers of acute COVID-19 severity such as hospitalization/ICU admission and related clinical features), and (3) concurrent correlates measured at baseline in the postacute phase (eg, patient-reported outcome measures and biomarkers at T0). Antecedent and periacute factors will be interpreted as candidate determinants, whereas concurrent measures will be interpreted primarily as phenotypic correlates differentiating Long COVID from recovered controls.

#### Analysis of Populations and Data Structure

The primary analytical dataset will consist of 1:1 matched pairs of Long COVID cases and recovered controls, with matching performed without replacement on sex/gender (exact), age (±3 years), and date of acute COVID-19 diagnosis (±30 days), and a retained pair_id. Data will be organized (1) in wide format for descriptive summaries by group and time point and (2) in long format (1 row per participant per time point) for longitudinal models incorporating T0 and T1.

##### Objective 1 (Primary): Identify Factors Associated With Long COVID Status (Case vs Recovered Control)

Consistent with the study hypothesis that biopsychosocial characteristics, biomarkers, lifestyle factors, and comorbidities are associated with symptom persistence, the primary inferential analyses will model Long COVID status as the dependent variable.

As an initial descriptive step, we will summarize within-pair distributions and conduct paired comparisons as appropriate (eg, McNemar tests for binary variables; paired *t* tests, or Wilcoxon signed-rank tests for continuous variables). These comparisons will be used to describe patterns of differences and will not be used as the sole criterion for variable inclusion in multivariable models.

Adjusted associations with Long COVID status will be estimated using conditional logistic regression stratified by pair_id (matched sets), reporting adjusted odds ratios with 95% CIs. Model building will follow a prespecified, interpretable structure. A core adjustment set defined a priori will be included to address confounding not already controlled by matching (eg, acute disease severity indicators such as hospitalization/ICU admission where available, vaccination status/epoch if captured, and comorbidity burden). Candidate predictors will then be entered in clinically coherent blocks (eg, lifestyle/psychosocial block and biomarker block) to support interpretation of how associations change after adjustment for confounding and correlated predictors. Continuous predictors will be assessed for appropriate functional form (eg, linearity in the logit); flexible specifications (eg, splines) or clinically meaningful categories will be used when needed. Collinearity will be assessed (eg, variance inflation), and influential observations will be examined using standard diagnostics.

Biomarkers (routine laboratory tests, inflammatory markers including CRP and IL-6, cytokine panel, quantitative antispike immunoglobulin G, and viral reactivation serologies such as EBV/cytomegalovirus/HSV-1) will be treated as candidate correlates of Long COVID status. Continuous biomarkers will be summarized with appropriate transformations if skewed (eg, log transformation), and extreme values will be checked for plausibility and laboratory flags prior to analysis.

To assess whether associations differ across clinically relevant strata, interaction terms (eg, predictor × sex/gender; predictor × acute severity; predictor × cohort type) will be evaluated where justified by the objective and sample size; interactions will be reported as stratum-specific estimates with uncertainty.

##### Objective 2: Characterize the Long COVID Cohort and Compare Profiles With Matched Recovered Controls

Baseline (T0) characteristics will be reported for cases and matched controls using paired summaries (within-pair differences where informative) and standard descriptive statistics (means [SD] or medians [IQR] for continuous variables and frequencies and percentages for categorical variables). Distributional assumptions will be checked (eg, Shapiro-Wilk for guidance, complemented by graphical inspection).

##### Objective 3: Evaluate Symptom and Outcome Evolution From T0 to T1

Longitudinal changes in continuous secondary outcomes (eg, questionnaire scores, cognitive test metrics, and selected continuous biomarkers) will be modeled using mixed-effects models including fixed effects for group (case vs control), time (T0 vs T1), and the group × time interaction to estimate differential change over time, with random intercepts for participants and appropriate correlation structures. Binary/ordinal outcomes will use generalized mixed-effects models with appropriate links (logit for binary; ordinal models for ordered scales where applicable). Primary longitudinal contrasts will be (1) within-group change and (2) between-group difference in change (interaction term), each reported with 95% CIs.

#### Exploratory Prediction/Risk Profiling (Secondary, Hypothesis-Generating)

To complement explanatory regression, predictive models will be developed to explore multivariable risk profiles. Random forest models will be used to rank variable importance and detect nonlinearities/interactions, followed by parsimonious regression-based models for interpretability. Model performance will be evaluated with internal validation (eg, k-fold cross-validation), reporting discrimination (area under the curve) and classification metrics; matched pairs will be kept together within the same resampling fold to avoid leakage across training/testing splits.

#### Missing Data, Multiplicity, and Sensitivity Analyses

Missingness will be summarized by variable and time point and assessed for patterns. If missingness is nontrivial for key predictors/outcomes, multiple imputation will be considered for analyses where assumptions are plausible; complete-case analyses will be presented as sensitivity checks. For families of secondary outcomes (multiple correlated questionnaire and biomarker measures), multiplicity control will be applied using a false discovery rate approach (eg, Benjamini-Hochberg) and reported alongside unadjusted estimates to preserve interpretability. Sensitivity analyses will include assessment of incomplete matching and, where necessary, inclusion of unmatched participants with regression adjustment for matching variables. Additional sensitivity analyses will evaluate robustness to alternative model specifications (eg, excluding highly collinear predictors and alternative transformations of skewed biomarkers) and to cohort type (retrospective vs prospective).

### Ethical Considerations

The study was approved by the Clinical Research Ethics Committee of Aragón, Spain (Comité de Ética de la Investigación Clínica de Aragón; PI21/278) and will be conducted in accordance with the Declaration of Helsinki and applicable local regulations. The collection and processing of personal data will comply with the GDPR (European Union) 2016/679 and the Spanish Organic Law 3/2018 of December 5 on the protection of personal data and guarantee of digital rights.

Biological samples and associated data will be managed through the Biobank of the Aragón Health System (National Biobank Register B.0000873; PT20/00112), integrated within the Instituto de Salud Carlos III (ISCIII) Biobanks and Biomodels Platform. Samples and data will be handled according to approved standard operating procedures and under the oversight and authorization of the relevant ethics and scientific committees and will be used exclusively for the purposes described in the approved protocol.

All participants will provide written informed consent prior to any study procedures, including specific consent for biobanking where applicable. Signed consent forms will be securely archived by the principal investigator. No participant compensation is planned due to limited funding.

### Privacy and Confidentiality

Participants will be assigned a unique internal study code at enrollment. Direct identifiers will be stored separately from research data, and the reidentification key linking identifiers to study codes will be accessible only to the principal investigator and stored in a restricted, encrypted location. Analytical datasets will be deidentified (coded) and will not include direct identifiers. Data will be collected and managed using secure electronic data capture systems with role-based access controls and audit trails and stored on secure institutional servers with restricted access. Data transfers, if required, will occur only via encrypted channels. Study results will be reported in aggregate form to minimize the risk of reidentification.

## Results

### Study Status, Approvals, and Registration

ARALongCOV is a registered observational study (ISRCTN27312680) with ethics approval from the Clinical Research Ethics Committee of Aragón (PI21/278). The study is conducted under GDPR and applicable Spanish data protection legislation, with biospecimens and associated data managed through the Biobank of the Aragón Health System (PT20/00112) following approved standard operating procedures. Funding was provided by the ISCIII through project PI22/01070 (cofounded by the European Union) for the period 2023-2027.

### Timeline and Data Collection Milestones

The study combines (1) a retrospective cohort providing long-term follow-up (enabling evaluation of symptom persistence for up to 3 years) and (2) a prospective cohort enrolling newly identified Long COVID cases with follow-up assessments after baseline. Baseline assessments (T0) were initiated in late 2022/early 2023, and follow-up assessments (T1) are scheduled for late 2025/early 2026 for the earliest enrolled participants. Study assessments are conducted at 2 time points (baseline [T0] and follow-up [T1]) using the same instruments and standardized procedures to support longitudinal comparisons. Data collection is organized into 2 appointments per assessment wave, including fasting blood sampling for routine laboratory analyses and completion of online questionnaires via REDCap.

### Recruitment and Follow-up Progress

The planned recruitment target is approximately 400 participants, exceeding the minimum analyzable sample size of 155 per group derived from power considerations for detecting moderate associations in the matched case-control framework. As of February 2026, a total of 200 participants have been enrolled (n=100 Long COVID; n=100 recovered controls) and have completed T0 assessments; T1 assessments are scheduled for late 2025/early 2026.

### Planned Reporting of Findings

Primary analyses will compare matched Long COVID and recovered participants using methods that account for the paired design (eg, conditional logistic regression with pair strata) and will evaluate longitudinal change using mixed-effects models including group, time, and group × time interaction. The first manuscripts reporting baseline comparisons and longitudinal outcomes are planned for submission in the second half of 2026, followed by mechanistic/biomarker and predictive modeling outputs.

## Discussion

### Overview

In this protocol, we describe a longitudinal matched case-control study design that combines retrospective and prospective data collection to investigate the multifaceted determinants of Long COVID in adult populations; such mixed longitudinal approaches have proven useful for characterizing recovery trajectories and for linking clinical phenotypes with immune and proteomic signatures in prior cohort work [3,48,49]. Our design explicitly aims to overcome limitations of single-dimension studies by integrating clinical, functional, and laboratory domains within a single, longitudinal framework to delineate temporal relationships among symptoms, objective physiologic measures, and circulating biomarkers and thereby to identify candidate mechanistic pathways and potential intervention targets [10,14,18].

### Strengths of the Study

This is a longitudinal matched case-control (1:1 matching) design. The prospective 3-year follow-up combined with retrospective characterization permits within-participant trajectory analysis and between-group comparisons that are less susceptible to the inferential constraints of purely cross-sectional or clinic-based samples [50].

The multimodal phenotyping and biospecimen through systematic collection of validated patient-reported instruments, functional measures, and comprehensive biomarker panels enhance the potential to identify biologically coherent PASC subtypes and to relate subjective symptoms to measurable biological perturbations [10,14,49,51].

In particular, including a matched recovered control group reduces confounding by age, sex, and calendar time (including variant and vaccination epoch) and helps distinguish sequelae specifically attributable to prior SARS-CoV-2 infection from those related to general post-illness or secular effects [5,18,24].

The use of machine learning and multivariable modeling (eg, random forests for variable importance and receiver operating characteristic analyses for predictive performance) enables exploration of high-dimensional interactions [52].

### Limitations

Despite these strengths, several important limitations merit emphasis. First, the targeted sample size of roughly 400 participants may limit power to detect associations for low-prevalence outcomes and to perform highly stratified subgroup analyses or fully corrected high-dimensional biomarker discovery without external validation, a limitation recognized in therapeutic and mechanistic PASC research [3,23]. Second, geographic confinement to a single region (Aragón, Spain) may reduce external validity because regional differences in variant circulation, vaccination patterns, sociodemographic composition, and health care delivery can influence both exposure and postacute outcomes; this limitation underscores the rationale for multicenter and multinational replication [29]. Third, recruitment through patient associations, health-service registries, and clinical referrals risks selection bias because individuals with persistent symptoms or heightened health care engagement may be overrepresented, an issue documented across online and clinic-recruited PASC cohorts [53]. Fourth, retrospective self-report carries the potential for recall and misclassification bias, and longitudinal attrition can induce informative censoring if participants who drop out differ systematically from those retained, both of which are well-recognized challenges in longitudinal PASC investigations [4,48,50,54].

### Future Research Directions

This protocol serves as a foundation for broader etiologic and interventional efforts. Key priorities for subsequent work include (1) expansion to multicenter/multinational networks to improve power and generalizability and to permit pooled analyses across variable systems and variant epochs [29], (2) harmonization of phenotyping and biospecimen protocols across cohorts to enable external validation and meta-analytic biomarker discovery [3,12,19], (3) incorporation of objective physiologic and imaging modalities (eg, cardiac and pulmonary imaging, cardiopulmonary exercise testing, and ambulatory wearable sensors) to corroborate and contextualize self-reported symptoms and to identify organ-level dysfunctions amenable to targeted therapies [55], and (4) prolonged longitudinal follow-up beyond 1 year to capture late-emerging sequelae and to quantify long-term risks of incident cardiometabolic or autoimmune conditions that have been reported in some cohorts [13,56].

### Broader Implications

Systematic identification of clinical, biological, and psychosocial determinants of Long COVID has immediate implications for clinical care and public health. Clinically, evidence of differential risk and mechanism-specific subtypes can inform stratified follow-up algorithms, personalized rehabilitation plans, and selection criteria for interventional trials [3,57]. From the public health and systems perspective, robust longitudinal data are necessary to develop standardized case definitions, to guide resource allocation for post–COVID-19 care pathways and rehabilitation services, and to underpin evidence-based policy and clinical guidance [23,29].

### Conclusions

This longitudinal matched case-control study, with mixed retrospective-prospective protocol, is designed to generate multimodal, longitudinal evidence on clinical, biological, and psychosocial correlates of Long COVID, leveraging matched controls, standardized phenotyping, and modern analytic methods to identify reproducible markers of symptom persistence and potential targets for intervention [3-5,18,23,52]. The study acknowledges limitations related to sample size, geographic scope, and selection or information biases and therefore positions its findings as a basis for validation in larger, multicenter consortia and for informing longer-term follow-up studies and therapeutic trials [29]. Ultimately, integrated longitudinal investigations of the type described here are essential to reduce the long-term burden of SARS-CoV-2 infection through improved prevention, diagnosis, and management of PASC [3,5,6,58].

## Appendix

Multimedia Appendix 1 Summary of validated instruments, scoring, and psychometric properties.

Multimedia Appendix 2 Original peer reviewed protocol and grant.

## Abbreviations

CRP: C-reactive protein
DSM: Diagnostic and Statistical Manual of Mental Disorders
EBV: Epstein-Barr virus
FSS: Fatigue Severity Scale
GDPR: General Data Protection Regulation
HSV-1: herpes simplex virus type 1
ICC: intraclass correlation coefficient
ICU: intensive care unit
IL-6: interleukin-6
IPAQ: International Physical Activity Questionnaire
ISCIII: Instituto de Salud Carlos III
ISRCTN: International Standard Randomized Controlled Trial Number
MEDAS: 14-item Mediterranean Diet Adherence Screener
pair_id: pair identifier
PASC: postacute sequelae of SARS-CoV-2 infection
PCFS: Post–COVID-19 Functional Status Scale
PCS: Pain Catastrophizing Scale
PHQ-9: Patient Health Questionnaire-9
PREDIMED: Prevención con Dieta Mediterránea
PSQI: Pittsburgh Sleep Quality Index
REDCap: Research Electronic Data Capture
SF-12: 12-item Short Form Health Survey
SF-36: 36-item Short Form Health Survey
STROBE: Strengthening the Reporting of Observational Studies in Epidemiology
T0: baseline assessment time point
T1: follow-up assessment time point
WHO: World Health Organization

## Data Availability

Because the study includes personal health data collected under GDPR and Spanish data protection law and involves biobank-linked samples, individual-level participant data cannot be made openly available without compromising confidentiality. A reproducibility package (study protocol, data dictionary/codebook, analysis plan, and analysis code/scripts) will be deposited in Zenodo; the public record will include a DOI and versioning to support transparent reuse. Access to deidentified individual-level datasets may be considered for bona fide research purposes on reasonable request and subject to (1) approval by the relevant ethics and/or scientific committees and biobank governance bodies, (2) a signed data use agreement, and (3) compliance with applicable legal requirements.
