# Supplementary material for: Differential Factors Associated With the Presence of Persistent Symptoms in Individuals Diagnosed With Long COVID: Protocol for a Longitudinal Matched Case-Control Study
Source: JMIR Res Protoc. 2026 Mar 24;15:e67133. doi: 10.2196/67133 (PMC13058537; doi:10.2196/67133)
Supplement: Multimedia Appendix 1 [file resprot_v15i1e67133_app1.docx]

**Table 2.** *Summary of validated instruments used in the study.*

| **Domain** | **Instrument (language)** | **Construct & recall period** | **Items / format** | **Scoring & interpretation** | **Measurement properties (key points)** | **Ref** |
| --- | --- | --- | --- | --- | --- | --- |
| Health-related quality of life | SF-12 Health Survey (Spanish) | HRQoL across 8 domains; typical recall as per SF instruments | 12 items; yields PCS and MCS summary scores | Norm-based PCS/MCS; higher = better HRQoL | Spanish evidence supports construct validity and adequate metric performance; SF-12 items explain ~91% of variance of SF-36 summary components; component reliability typically in acceptable-to-good range (≈0.78–0.85) | [42] |
| Physical activity | International Physical Activity Questionnaire (IPAQ) (Spanish) | Physical activity over **last 7 days** | Self-report; time in activity intensities; permits MET-min/week | Output as MET-min/week and categorical activity levels; higher = more activity | Spanish data show strong reproducibility (test–retest Spearman ρ≈0.81; ICC≈0.74); criterion validity vs accelerometry modest (ρ≈0.27) and higher vs activity diaries (ρ≈0.38), consistent with self-report PA measures | [43] |
| Diet | MEDAS 14-item Mediterranean Diet Adherence Screener (Spanish) | Mediterranean diet adherence; captures dietary targets (habitual pattern) | 14 items checklist | Total score **0–14**; higher = greater adherence | As a dietary pattern screener (not a unidimensional psychometric scale), performance is supported mainly by external validity (associations with relevant health/anthropometric indices in PREDIMED) | [44] |
| Sleep | Pittsburgh Sleep Quality Index (PSQI) (Spanish) | Sleep quality over **previous month** | 19 items; 7 component scores + global score | Global score **0–21**; higher = worse sleep; common threshold >5 indicates poor sleep quality | Original PSQI widely used in clinical/research settings; Spanish clinimetric evaluation supports use, with internal consistency around α≈0.81 and evidence of diagnostic performance (threshold-dependent sensitivity/specificity) | [45,46] |
| Depression | Patient Health Questionnaire-9 (PHQ-9) (Spanish) | Depressive symptoms over **previous 2 weeks** | 9 items; 0 (“not at all”)–3 (“nearly every day”) | Total **0–27**; higher = more severe depression; common severity bands: 5/10/15/20 | Spanish primary care validation shows strong internal consistency (eg, ω≈0.89) and good screening performance for major depressive disorder depending on cut-point/reference | [47] |
| Anxiety | Generalized Anxiety Disorder-7 (GAD-7) (Spanish) | Anxiety symptoms over **previous 2 weeks** | 7 items; 0–3 response options | Total **0–21**; higher = more severe anxiety; common severity bands: 5/10/15 | Spanish adaptation shows excellent internal consistency (α≈0.94) and strong short-term stability (test–retest ICC≈0.84), with evidence of concurrent validity via correlations with anxiety/disability measures | [48] |
| Functional status (post-COVID) | Post-COVID-19 Functional Status Scale (PCFS) (Spanish; web-based) | Functional limitations; ordinal grade (current functioning) | Single ordinal grade (self-completed web form via REDCap) | Grade **0–4** (0 = no limitations; 4 = severe limitations); higher = worse function | Ordinal grade (internal consistency not applicable). Spanish version shows adequate construct validity; **web-based** form shows substantial test–retest reliability (kappa≈0.63) and high agreement vs paper (≈88% agreement) | [49,50] |
| Cognition: attention/executive control | Stroop Color and Word Test | Selective attention, inhibitory control (executive function); immediate performance | Performance-based; standardized conditions; interference index | Standard condition scores + interference index; higher impairment reflected by poorer performance per scoring method | Manualized test with extensive normative/clinical use; reliability supported through standardized administration and established scoring procedures | [51] |
| Cognition: processing speed/attention | Symbol Digit Modalities Test (SDMT) | Processing speed, sustained attention; immediate performance | Timed substitution task | Standard SDMT score (per manual); lower = worse processing speed/attention | Manualized test with extensive normative use; standardized administration enhances reproducibility across waves | [52] |
| Cognition: visuoconstruction & visual memory | Rey–Osterrieth Complex Figure | Visuoconstruction (copy) and visual memory (recall); immediate and delayed depending on protocol | Performance-based drawing task | Copy accuracy and recall scores using standardized scoring; lower = worse | Widely used with standardized scoring procedures; reproducibility supported by trained administration and consistent scoring rules | [53] |
| Pain-related cognition | Pain Catastrophizing Scale (PCS) (Spanish) | Catastrophizing: rumination, magnification, helplessness (current/typical) | 13 items (typically 0–4 response scale) | Total **0–52**; higher = greater catastrophizing | Original scale has robust psychometric development; Spanish version demonstrates excellent internal consistency (α≈0.94) and strong test–retest reliability (ICC≈0.88) | [54,55] |
| Fatigue | Fatigue Severity Scale (FSS) (Spanish use) | Fatigue severity/impact on functioning (typical/current) | 9 items; typically rated 1–7 | Mean score **1–7** (or summed per convention); higher = more severe fatigue | Original scale established in chronic disease; in post-COVID recovery settings shows excellent internal consistency (α≈0.96) and expected construct validity (eg, correlations with HRQoL and depressive symptoms) | [56,57] |
