# Supplementary material for: Differential Factors Associated With the Presence of Persistent Symptoms in Individuals Diagnosed With Long COVID: Protocol for a Longitudinal Matched Case-Control Study
Source: JMIR Res Protoc. 2026 Mar 24;15:e67133. doi: 10.2196/67133 (PMC13058537; doi:10.2196/67133)
Supplement: Multimedia Appendix 2 [file resprot_v15i1e67133_app2.pdf]

Machine Translated by Google
CSV Validation

GOBIERNO DE ESPAÑA | MINISTERIO DE CIENCIA E INNOVACIÓN | Instituto de Salud Carlos III

HEALTH RESEARCH PROJECTS EVALUATION REPORT

GENERAL INFORMATION

RECORD	PI22/01070
PRINCIPAL INVESTIGATOR	MAGALLON BOTAYA, ROSA MARIA
QUALIFICATION	Analysis of differential factors associated with the presence of persistent symptoms in people diagnosed with COVID-19: Case-control studies
REALIZATION CENTER	Aragon Health Research Institute
REQUESTING CENTER	Aragon Health Research Institute Foundation
TECHNICAL COMMISSION OF ASSESSMENT	Epidemiology, public and occupational health

EVALUATION REPORT (max. 100 points)

SCORES

ASSESSMENT EQUIPMENT 24.00 RATING PROJECT 60.00 EMERGENT 0.00 DISABILITY 0.00 TOTAL 84.00

A) RESEARCH TEAM ASSESSMENT (max. 30 points).

1st.- Scientific history (projects, publications, clinical practice guidelines and patents) of the PI and the group in relation to the subject of the project. (From 0 to 8 points).

8.00

2nd Quality and leadership in international R&D&I programs. (From 0 to 4 points).

0.00

3rd.- Training capacity of the PI and the team: supervision of Doctoral and Master's theses and funding of HR in competitive calls. (From 0 to 4 points).

4.00

4th.- Preliminary results obtained in the scope of the proposal. (From 0 to 4 points).

4.00

5th.- Proven track record or potential of the principal investigator and the research team in the development of R&D&I activities within the framework of the AES. (From 0 to 10 points).

8.00

Score: TEAM 24.00

FIRMADO
This CSV

Machine Translated by Google

CSV Validation

B) Project evaluation (max 70 points)

1st.- Quality: Assessment of the hypothesis and the level of competence regarding the current state of knowledge on the subject; novelty, originality and innovation of the proposal; clarity and specificity in the formulation of the objectives; methodological suitability to the proposed objectives, and proposal for the Data Management Plan (DMP). (From 0 to 15 points).

12.00

2. Feasibility: Suitability of the team to the proposal; work plan, task distribution and timeline; available infrastructure and management capacity; suitability of the requested budget to the project objectives. Complementarity of the participating research teams and benefits of coordination. (From 0 to 15 points).

14.00

3rd Relevance, interest, applicability and transferability of the project: health, economic and social impact. (From 0 to 30 points).

**• Impact on health (From 0 - 20 points)**	17.00
**• Economic Impact (0-5 points)**	3.00
**• Social Impact (0 - 5 points)**	4.00

4. Suitability and relevance of the proposal to clinical and/or translational research. (From 0 to 10 points)

10.00

**Emerging IP and meets priority criteria**	NO
**Assessment of the quality and viability of the project >= 20 points**	YEAH
**Emerging IP Increase**	0.00
**IP / CoIP with a degree of disability ÿ 33%**	NO

Score: PROJECT 60.00

FIRMADO

ThisECSV

Machine Translated by Google

CSV Validation

GOBIERNO DE ESPAÑA | MINISTERIO DE CIENCIA E INNOVACIÓN | Instituto de Salud Carlos III

HEALTH RESEARCH PROJECTS EVALUATION REPORT

ASSESSMENT

Project Assessment

Project submitted by a well-established group with research experience in the AES and in the subject of the proposal. The group is large and multidisciplinary, although its international leadership capacity is not specified.

The project is relevant, feasible, and has clinical impact. Although some scientific evidence already exists on the topic, the project presents original elements with potential applicability in the SPS, addressing an emerging and prevalent issue. Hypothesis, objectives and methodology would have benefited from greater clarity.

The budget is adequately justified, although its line items could be adjusted slightly.

TOTAL Score 84.00

Is this project subject to scientific evaluation nine months after the start of the implementation period? NO

Is the beneficiary urged to reformulate their application during the appeals period to adjust the commitments and conditions to the grant awarded? NO

EVALUATOR ID: 5655B

The personal data appearing in this document will only be used for the purposes set out in the corresponding Call for Proposals or for automated processing for statistical purposes with the guarantees provided for in Regulation (EU) 2016/679 of the European Parliament and of the Council of 27 April 2016 on the protection of natural persons with regard to the processing of personal data and on the free movement of such data and repealing Directive 95/46/ EC (General Data Protection Regulation) and in Organic Law 3/2018, of 5 December, on the Protection of Personal Data and the guarantee of digital rights.

FIRMADO

This CSV
